# Supplementary figures and images for: Exploring the role of the various methionine residues in the Escherichia coli CusB adapter protein
Source: PLoS One. 2019 Aug 29;14(8):e0219337. doi: 10.1371/journal.pone.0219337 (PMC6715271; doi:10.1371/journal.pone.0219337)

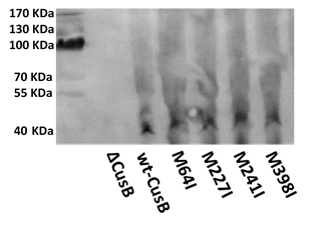

Supplement: S1 Fig — (TIF) [file pone.0219337.s001.tif]

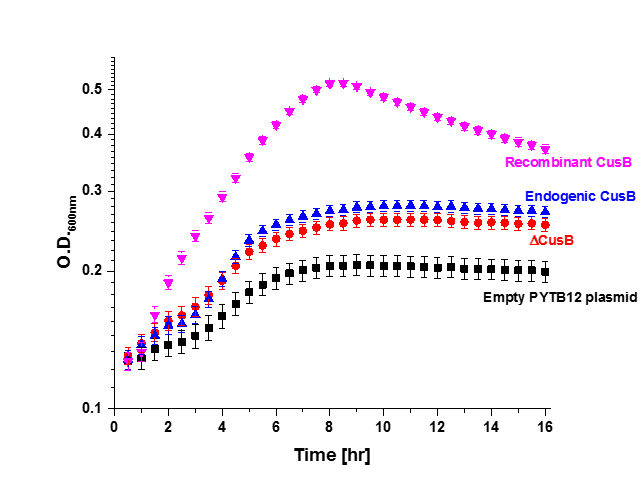

Supplement: S2 Fig — OD after 16 hr for recombinant CusB, endogenic CusB, ΔCusB and BL21 cells with empty PYTB12 plasmid. (TIF) [file pone.0219337.s002.tif]

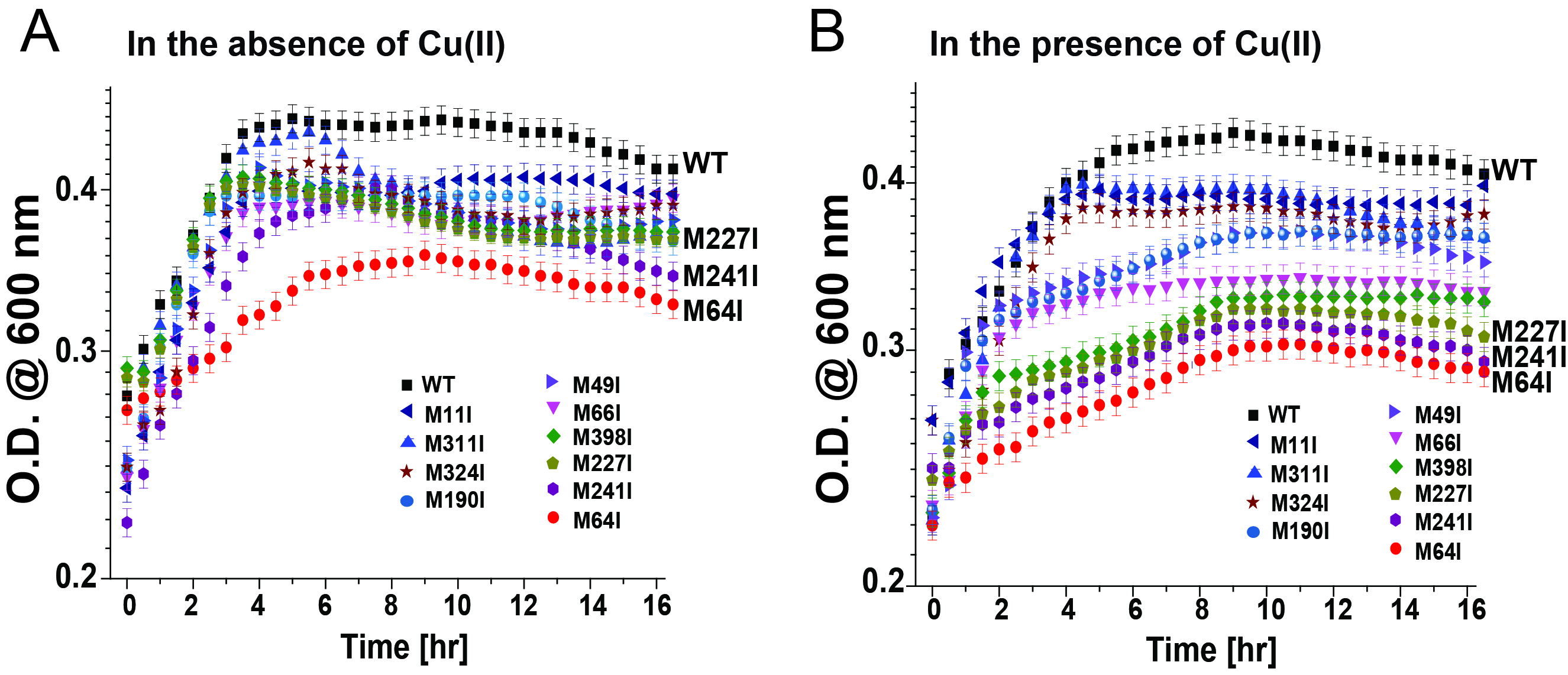

Supplement: S3 Fig — Cell growth rates for various CusB clones in the absence (A) and presence (B), respectively, of 3 μM Cu(II). (TIF) [file pone.0219337.s003.tif]

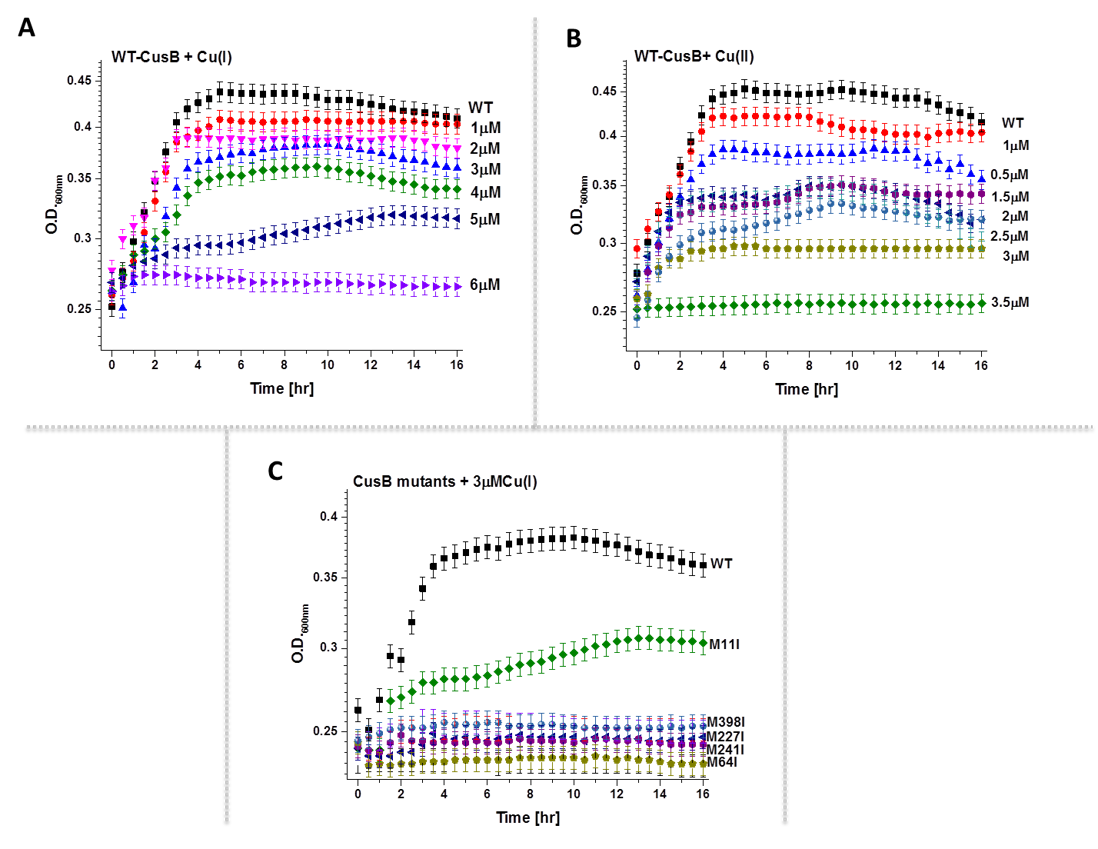

Supplement: S4 Fig — A. OD after 16 hr for native E. coli cells as affected by Cu(I) concentration. B. OD after 16 hr for ΔCusB cells affected by Cu(I) concentration. For ΔCusB cells, cell growth was terminated at a lower Cu(II) concentration. C. OD after 16 hr for ΔCusB-mutant cells as affected by Cu(I) concentration. (TIF) [file pone.0219337.s004.tif]

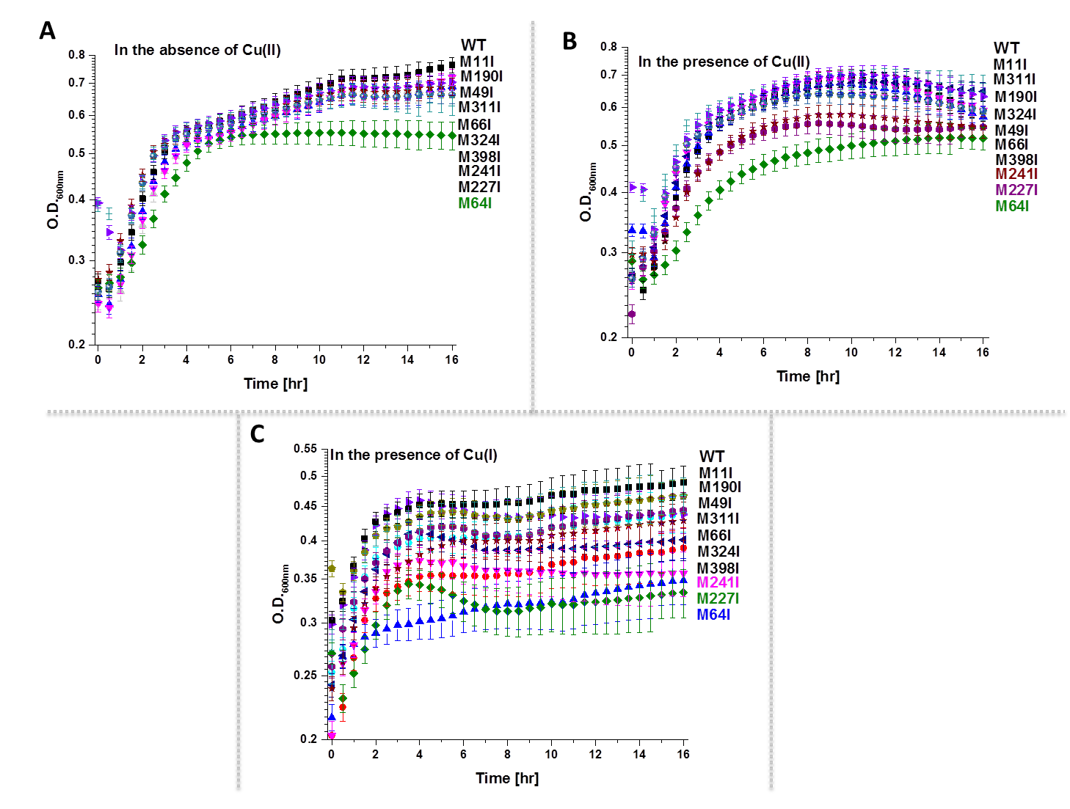

Supplement: S5 Fig — A. OD after 16 hr for native E. coli cells grown in LB medium. B. OD after 16 hr for ΔCusB cells grown in LB medium as affected by 3mM Cu(II). C. OD after 16 hr for ΔCusB cells grown in LB medium as affected by 3mM Cu(I). (TIF) [file pone.0219337.s005.tif]

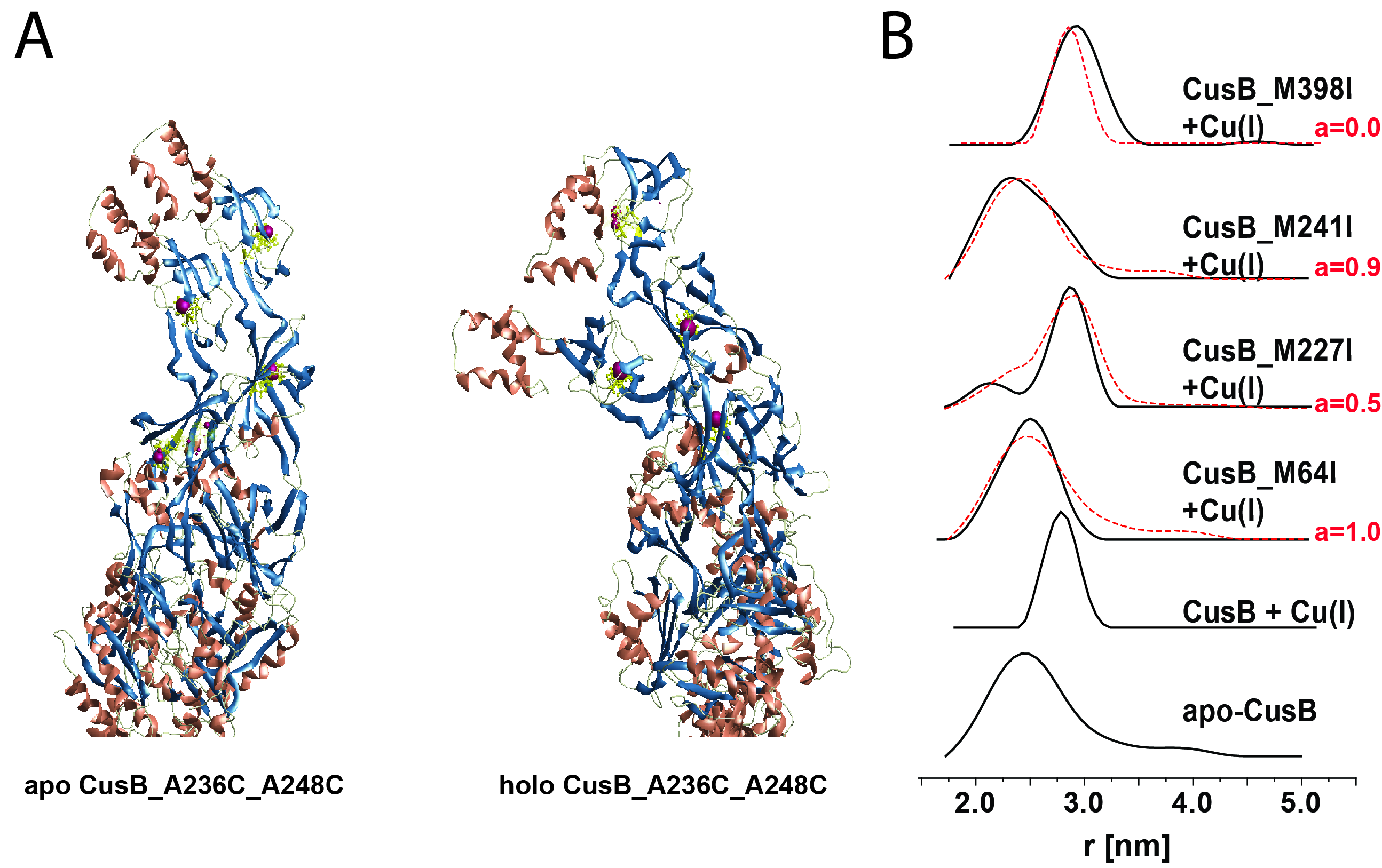

Supplement: S6 Fig — A. Spin-label positions and orientations attached to CusB_A236C_A248C in apo and holo states. B. The distance distribution obtained and shown in Fig 3B. The red dotted lines corresponds to the linear composition a*f(r)+(1-a)*g(r); where f(r) is apo CusB distance distribution and g(r) is CusB+Cu(I) distance distribution. (TIF) [file pone.0219337.s006.tif]
