# Supplementary material for: Exploring the role of the various methionine residues in the Escherichia coli CusB adapter protein
Source: PLoS One. 2019 Aug 29;14(8):e0219337. doi: 10.1371/journal.pone.0219337 (PMC6715271; doi:10.1371/journal.pone.0219337)
Supplement: S1 File — (PDF) [file pone.0219337.s007.pdf]

Materials and Methods

CusB cloning, expression, and purification

We described the complete procedure previously (1). The CusB construct was expressed in BL21 cells, which were grown to an optical density of 0.6–0.8 at 600 nm and were induced with 1 mM isopropyl-β-d-thiogalactopyranoside (Calbiochem, San Diego, CA, USA) for 16 hr at 18°C. Cells were harvested by centrifugation [at 8000 × g]; the pellets were subjected to three freeze-thaw cycles and then were resuspended in lysis buffer. After sonication, the cells were centrifuged [at 12000 × g] and the soluble fraction of the lysate was passed through a chitin-bead column to enable the CusB fusion to bind to the resin via its chitin-binding domain. The resin was then washed with 30 column-volumes of lysis buffer. To induce intein-mediated cleavage, the beads were incubated in 50 mM dithiothreitol (DTT), 25 mM NaH2PO4, and 150 mM NaCl at pH 8.9 for 40 hr at room temperature. CusB was collected in elution fractions and analyzed by using silver-stained sodium dodecylsulfate polyacrylamide gel electrophoresis (SDS-PAGE) (10% glycine) (2). An identical protocol was used for mutant formation.

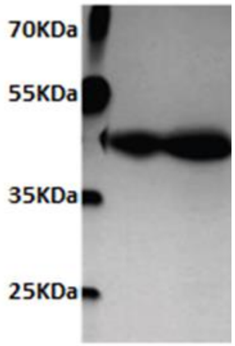

Fig A: ***SDS-PAGE gel:*** **Glycine 10% SDS-PAGE gel of CusB_WT at various concentrations (0.1 mM and 0.3 Mm), to confirm the expression and purification of CusB.**

CusB spin labeling

CusB was labeled by an initial overnight incubation with 10 mM DTT. The DTT was removed and S-(2,2,5,5-tetramethyl-2,5-dihydro-1H-pyrrol-3-yl) methyl methane sulfonothioate (MTSSL, TRC) dissolved in dimethyl sulfoxide was added to the protein. Free spin label was removed with a Microsep Advanced Centrifugal Device (Pall, Port Washington, NY, USA; ref. no. MCO003C41) applied to samples of up to 5 mL in lysis buffer, with a 3-kDa molecular weight cutoff . A sample of the running washing buffer was taken for Continuous-Wave (CW) EPR measurement at room temperature (RT), and no free-spin EPR signal was observed. The concentration was determined with a Lowry assay (3). The final concentration of spin-labeled CusB protein was 0.01 – 0.02 mM.

Addition of Cu(I) ion to protein solution

Cu(I) (tetrakis (acetonitrile) copper(I) hexafluorophosphate (Sigma-Aldrich, St. Louis, MO, USA)) was added to the protein solution under nitrogen gas to maintain inert anaerobic conditions. No Cu(II) EPR signal was observed at any time. A Cu(I):CusB ratio of 3:1 was used for all EPR measurements.

Electron Paramagnetic Resonance (EPR) spectroscopy

A constant-time four-pulse double electron-electron resonance (DEER) experiment with pulse sequence π/2(vobs)-τ1-π(vobs)-t′−π(vpump)-(τ1 + τ2 − t′)-π(vobs)-τ2(vobs)-τ2-echo was performed at (50 ± 0.5 K) on a Q-band Elexsys E580 equipped with a 2-mm probe head; bandwidth, 220 MHz. A two-step phase cycle was applied to the first pulse. The echo was measured as a function of t′, and τ2 was kept constant to eliminate relaxation effects. The pump pulse frequency was set to the maximum of the EPR spectrum and the observer pulse frequency was set 60 MHz higher than that of the pump pulse. The observer π/2 and π pulses, as well as the π pump pulse, had durations of 40 ns; the dwell time was 20 ns. The observer frequency was 33.82 GHz. The power of the 40-ns π-pulse was 20.0 mW. The parameter τ1 was set to 200 ns, and τ2 was set to 1200 ns. The repetition time was set to 12 ms, and 30 shots per point were applied. The samples were measured in 1.6-mm quartz capillary tubes (Wilmad-Labglass, Vineland, NJ, USA). The data were analyzed with the DeerAnalysis 2016 program and Tikhonov regularization (4,5). The regularization parameter in the L curve was optimized by examining the fit of the time-domain data and was found to be between 30 and 40. The modulation depth for the four-spin system was about 15%.

In-vitro measurements

Determining the Cu(I) dissociation constant

BCA (Bicinchoninic acid disodium salt hydrate; Sigma-Aldrich, St. Louis, MO, USA) and Cu(I) tetrakis (acetonitrile) copper(I) hexafluorophosphate (Sigma-Aldrich) solution was titrated with CusB and their UV-VIS spectra were recorded with a Chirascan spectrometer (Applied Photophysics, Surrey, UK) at RT. Measurements were carried out in a cell with a 1-mm optical path length. The Cu(I) concentration was 100 µM and the BCA concentration was 220 µM to ensure the absence of free Cu(I) from the solution. Spectra were recorded from 200 to 800 nm with a step size and bandwidth of 0.5 nm. The spectra were baselined according to the absorption value at 800 nm, which is zero.

To correlate between the concentration of BCA-Cu(I) and its absorbance at 562 nm, UV-Vis spectra were recorded for 200 µM BCA titrated with Cu(I)(6) (Fig B).

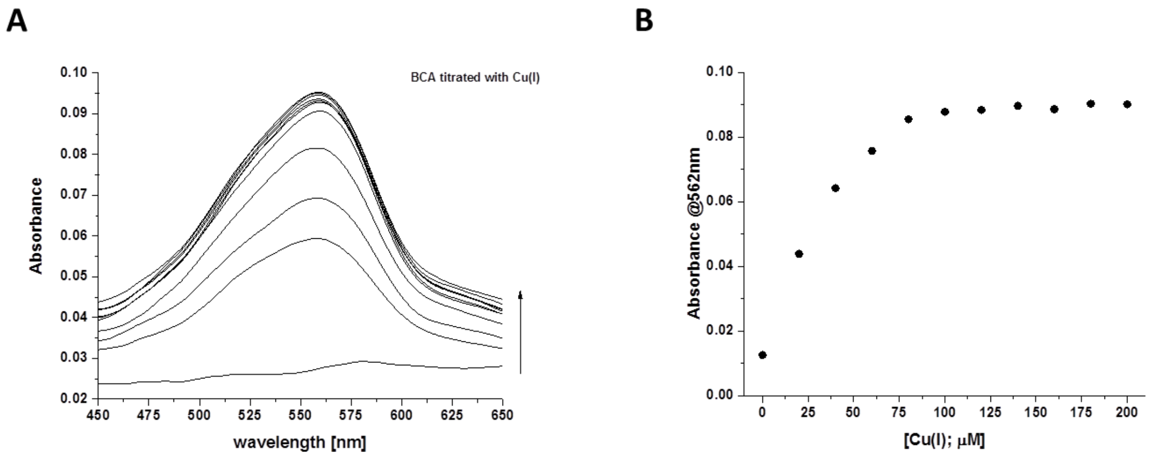

Fig B: UV-Vis absorption measurements. A. UV-Vis spectra for 200 µM BCA as affected by 100 µM Cu(I) titration. B. Absorbance at 562 nm for 100 µM Cu(I) as affected by BCA titration.

Fig C shows the absorption spectra for [Cu(I)–BCA_2] as affected by ∆CusB-mutants titration.

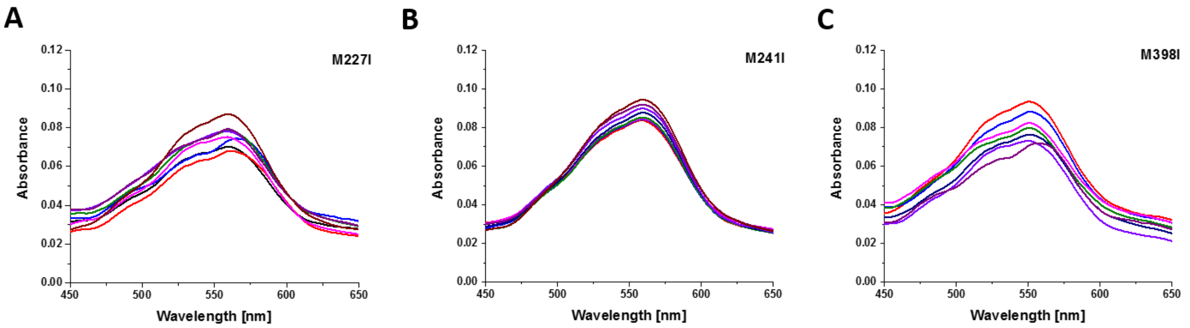

Fig C: UV-Vis absorption measurements. UV-Vis spectra for 200 µM BCA as affected by Cu(I) titration for: A. M227I; B. M241I; and C. M398I.

Tables A-C present the three repetitions for K_D values calculated for ΔCusB-mutants

Table A: K_D calculations for ΔCusB-mutants.

	[Cu(I)] µM	[BCA] µM	[protein] µM	Abs at 562 nm	[Cu(I)-BCA2]	[Cu(I)-protein]	**K_D [M];×10^{-14}**
without CusB	100	220	0	0.095	100.0	0	
WT	100	220	100	0.065	8.25	91.75	9.3
M64I	100	220	100	0.090	11.40	88.60	27.1
M227I	100	220	100	0.071	9.07	90.93	12.7
M241I	100	220	100	0.080	10.16	89.84	18.4
M398I	100	220	100	0.065	8.20	91.80	9.13

Table B: K_D calculations for ΔCusB-mutants.

	[Cu(I)] µM	[BCA] µM	[protein] µM	Abs at 562 nm	[Cu(I)-BCA2]	[Cu(I)-protein]	**K_D [M];×10^{-14}**
without CusB	100	220	0	0.095	100.0	0	
WT	100	220	100	0.063	8.00	92.00	8.45
M64I	100	220	100	0.091	11.52	88.50	28.1
M227I	100	220	100	0.079	9.97	90.03	17.3
M241I	100	220	100	0.081	10.25	89.75	19.0
M398I	100	220	100	0.064	8.10	91.90	8.78

Table C: K_D calculations for ΔCusB-mutants.

	[Cu(I)] µM	[BCA] µM	[protein] µM	Abs at 562 nm	[Cu(I)-BCA2]	[Cu(I)-protein]	**K_D [M];×10^{-14}**
without CusB	100	220	0	0.095	100.0	0	
WT	100	220	100	0.063	7.97	92.03	8.42
M64I	100	220	100	0.100	12.64	89.36	28.4
M227I	100	220	100	0.078	9.90	90.10	16.9
M241I	100	220	100	0.082	10.40	89.60	19.9
M398I	100	220	100	0.064	8.12	91.88	8.83

Circular Dichroism (CD) measurements

In order to verify that CusB mutants have no effect on the secondary structure of the protein, we used Circular dichroism (CD) measurements. CD measurements were conducted using a Chirascan spectrometer (Applied Photophysics). Measurements were performed in a 1-cm optical path length cell, and the spectra were recorded from 270 to 190 nm with a step size and a bandwidth of 0.5 nm. The CD signal was averaged for 10 s every 2 nm, 3 scans per sample.

Fig D presents CD spectra for various CusB mutants.

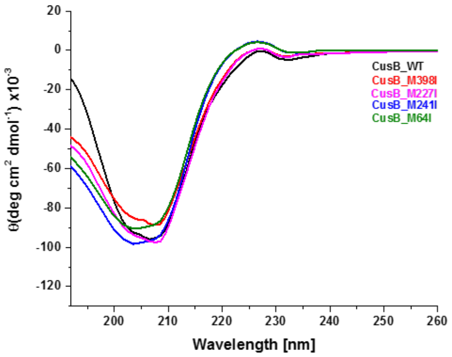

	%α-helix	%β-sheet	%Random Coil
WT-CusB	15.2±0.4	53.9±0.6	30.9±0.4
M64I	15.8±0.7	51.8±0.9	32.4±0.5
M227I	16.1±0.9	56.3±0.7	27.6±0.3
M241I	15.3±0.6	53.5±0.9	31.2±0.2
M398I	18.1±0.8	50.5±0.4	31.4±0.7

Fig D: CD spectra and analysis for CusB_WT, CusB_M64I, CusB_M227I and CusB_M241I

The CD spectra reveal that upon mutation the secondary structures of the protein is affected by less than 3%.

Western Blot

In order to compare the growth rate for the wt-CusB and its various mutants their protein expression level must be equivalent. WT-CusB and the various mutants were grown during 16 hours in LB medium. Pellets were suspended in SDS sample buffer, separated by SDS-PAGE and transferred to a polyvinylidene difluoride membrane using a transfer apparatus according to themanufacturer's protocols (Bio-Rad). After incubation with 3% BSA in TBST (10 mMTris, pH 8.0, 150 mM NaCl, 0.5% Tween 20) for 60 min, the membrane was incubated with antibodies against CusB (1:1000) at 4 °C overnight. The membrane was washed with TBST three times for 10 min and incubated with a 1:20000 dilution of peroxidase-conjugated anti-mouse antibody for 60 min. The blot was washed with TBST three times for 10 min and developed with the ECL system (Bio-Rad) according to manufacturer's protocols.

Cell experiments

CusB knock out (∆CusB)

∆CusB was carried out with the Quick & Easy E. Coli Gene Deletion Kit (Gene Bridges; version 2.4).

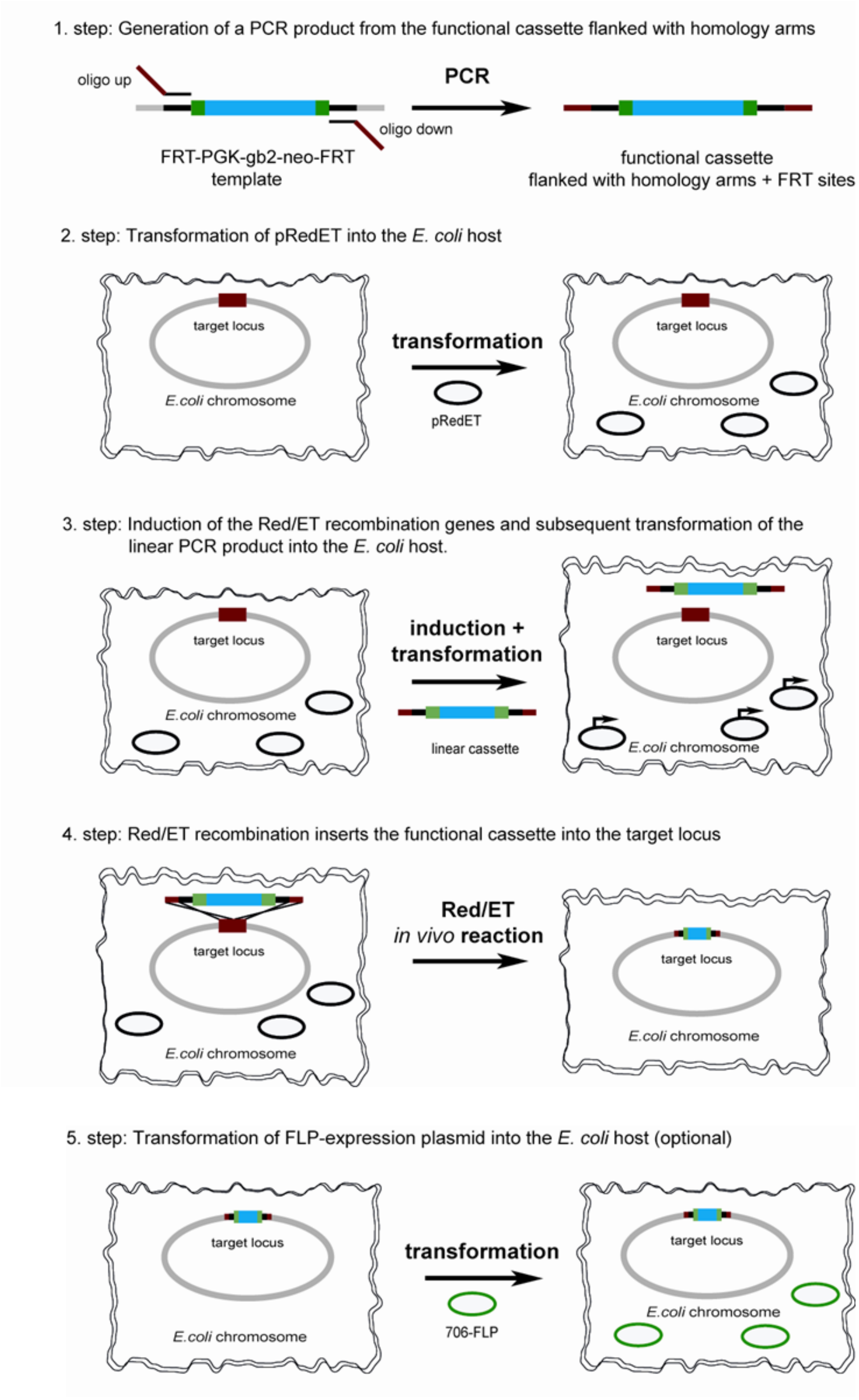

Fig E: Flowchart of the experimental outline for the targeted disruption of genes on the E. coli chromosome.

Generation of a PCR product from the functional cassette flanked with homology arms

The oligonucleotides were suspended in double distilled water at a final concentration of 10 µM. The PCR reaction was conducted following Table D.

To target the chromosome at the site of choice, it is necessary to incorporate short homology regions into the functional cassette carrying the selectable marker "Sm". This is done by designing two oligonucleotides for use in PCR amplification. Each oligonucleotide consists of two parts:

1. The first part is the homology region (see primer A/B not underlined) shared by the target molecule and the linear molecule. The homology regions are the 50 bp directly adjacent to either side of the insertion site. The exact sequences of the homology regions can be chosen freely, depending on the position on the target molecule to be modified.

2. The second part, usually 18 to 24 nucleotides long (23 nucleotides were selected see primer A/B underlined), primes the PCR amplification of the selectable marker from the provided template.

The primers selected for the procedure were as follows:
Primer A-
ATGAAAAAAATCGCGCTTATTATCGGCAGCATGATCGCGGGCGGTATTATAATTAACCCTCACTAAAGGGCGG
Primer B-
TCAATGCGCATGGGTAGCACTTTCAGAGCGCATCCGCTCCAGTGCGCCAGTAATACGACTCACTATAGGGCTC

Table D: PCR reaction conditions

ddW	18 µL
GoTaq® Green Master Mix, 2× (Promega, Madison, USA)	25 µL
Primer A (100µM)	3 µL
Primer B (100µM)	3 µL
FRT-PGK-gb2-neo-FRT PCR-template	1.0 µL

PCR profile: Initial denaturation step 5 min at 98 ºC; thirty cycles: 30 sec at 98 ºC, 30 sec at 57.5 ºC, 120 sec at 72 ºC; final elongation step 10 min at 72 ºC. (see Fig F)

PCR products were purified using Wizard® SV Gel and PCR Clean-Up System (Promega, Madison, USA).

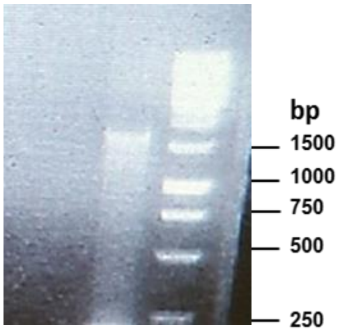

Fig F: 1% agarose gel indicating the generation of a PCR product from the functional cassette flanked with homology arms.

Transformation with Red/ET expression plasmid pRedET:

BL21 E. coli cells were streaked out on LB plates. Two colonies were inoculated in microfuge tubes containing 1mL LB medium. Incubation was done at 37 ºC overnight with shaking.

Two microfuge tubes containing fresh 1.4 mL LB medium were inoculated with 30 µL of overnight grown culture. The tubes were incubated for three hr at 37 ºC, shaking at 1000 rpm. Tubes were centrifuged for 30 sec at 11000 rpm in a cooled microfuge benchtop centrifuge (at 4 ºC). Pellets were suspended in 1 mL chilled double distilled water. Centrifugation and resuspension of the cells was repeated in volumes of 30 µL.

1 µL of Red/ET Recombination protein expression plasmid pRed/ET was added to cell pellets. Cells were transferred into chilled 1 mm electroporation cuvettes.

Electroporation was conducted at 1350 V, 10 µF, 600 Ω in an Eppendorf® Electroporator 2510.

Cells were transferred into 1 mL LB medium incubated at 30 °C for 70 min, shaking at 1000 rpm. Next, 100 µL cells were streaked out on LB plates containing tetracycline (3 µg/mL). E. coli cells + pRed/ET were streaked out on LB agar plates containing: **A**. tetracycline (3 µg/mL), **B**. tetracycline (3 µg/mL) + kanamycin (15 µg/ml), and **C**. kanamycin (15 µg/ml) as control experiments. Plates were incubated at 30 °C overnight wrapped in tinfoil. No colonies were found for plates **B** and **C**; however, 9 colonies were found on plate **A**.

Disruption of a chromosomal DNA fragment by the FRT-flanked PGK-gb2-neo cassette:

Colonies were picked from tetracycline plates and control plates and inoculated in a microfuge tube containing 1 mL LB medium + 3 µg/mL tetracycline. Tubes were incubated while shaking at 30 °C overnight. Next, 30 µL of overnight cultures were inoculated into four microfuge tubes containing 1.4 mL fresh LB medium and tetracycline (3 µg/mL), then incubated for 2 hr at 30 °C while shaking at 1100 rpm until O.D.600nm~0.3. Next, 50 µL of 10% L-arabinose solution were added to one the experiment's tubes and to a control tube, giving a final concentration of 0.3%. The remaining two tubes were left without induction as negative controls. All tubes were incubated at 37 °C, while shaking for 1 hr at 1100 rpm.

Three LB petri dishes inoculated with A. 2 µL PCR products, B. 4 µL PCR products, and C. without L-arabinose solution were streaked with cells and incubated at 37 °C overnight. Plate A presented 10 colonies, 1 colony was found for plate B, and no colonies could be detected for plate C.

Tubes were centrifuged for 30 sec at 11000 rpm in a cooled microfuge benchtop centrifuge (at 4 ºC). Pellets were suspended in 1mL chilled double distilled water. Centrifugation and resuspension of the cells were repeated in a volume of 30 µL. Next, 2 µL (400ng) of the linear FRT-PGK-gb2-neo-FRT fragment was added to the pellet to each of the two microfuge tubes (induced and uninduced). Pellets were pipetted into chilled electroporation cuvettes. In parallel, 2 µL from FRT-PGK-gb2-neo-FRT PCR-product was pipetted into each of the two tubes of the control. Electroporation was conducted at 1350 V, 10 µF, 600 Ω in an Eppendorf® Electroporator 2510. Cells were transferred into 1 mL LB medium incubated at 37 °C for 3 hr while shaking at 1000 rpm for recombination to occur. The whole cultures were spanned down for 30 sec and 900 µL of the supernatant were removed. Cells were resuspended in the remaining medium and streaked out onto LB agar plates containing kanamycin (15 µg/ml). Petri dishes were incubated at 37 °C overnight [the Red/ET Recombination protein expression plasmid (pRedET) disappears at 37 °C].

∆CusB cells were streaked out on petri dishes to obtain single colonies, which were then analyzed by PCR using:

1) Primer A and primer 2 (kit): 5'- CGAGACTAGTGAGACGTGCTAC-3'.

2) Primer B and primer 3 (kit): 5'- TATCAGGACATAGCGTTGGCTACC-3'.

3) Primer A and primer B.

PCR profile: Initial denaturation step 5 min at 98 ºC; thirty cycles: 30 sec at 98 ºC, 30 sec at 57.5 ºC, 120 sec at 72 ºC; final elongation step 10 min at 72 ºC. (See Fig G lanes 1-3 accordingly.)

∆CusB cells, recombinant CusB cells, and BL21 cells (endogenic CusB) were streaked out on a petri dish to obtain single colonies, which were then analyzed by PCR using:

CusB 5′: GTTGTACAGAATGCTGGTCATATGAAAAAAATCGCGCTTATTATCG

CusB 3′: GTCACCCGGGCTCGAGGAATTTCAATGCGCATGGGTAGC

PCR profile: Initial denaturation step 5 min at 98 ºC; thirty cycles: 30 sec at 98 ºC, 3.5 min at 70 ºC, 7 min at 72 ºC; final elongation step 10 min at 72 ºC. (See Fig G lanes 4-6 recombinant CusB, BL21, ∆CusB accordingly.)

For recombinant CusB (lane 4) and endogenic CusB (lane 5), a bend is presented at ~1250 bp indicating the presence of the CusB gene; as for **∆CusB (lane 6) no bend is formed, since the CusB gene is deleted.**

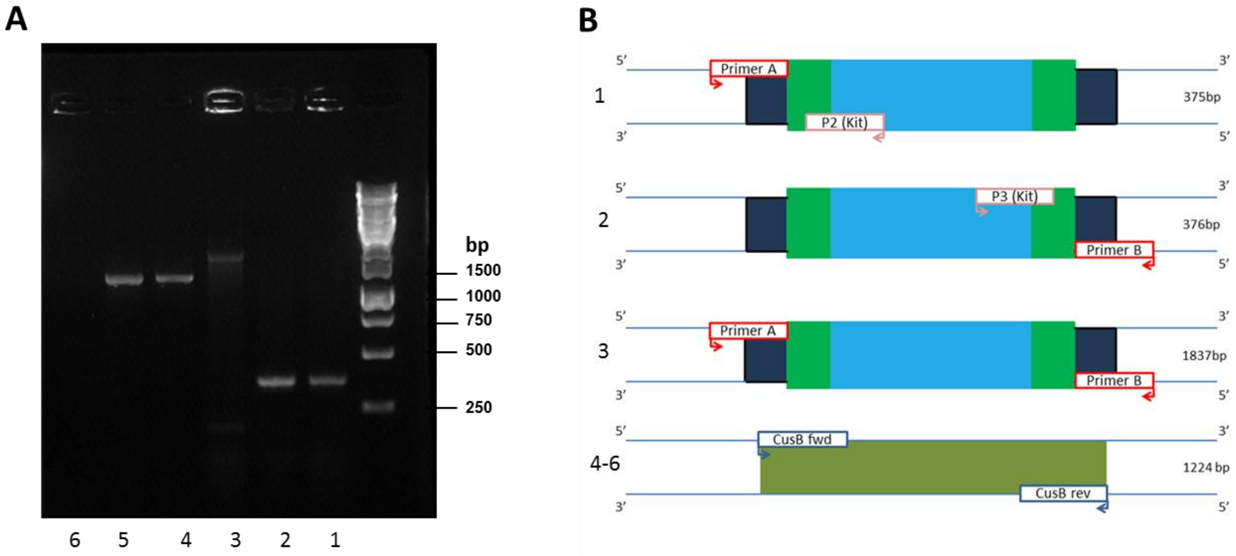

Fige G: A. 1% agarose gel, colony PCR for ∆CusB cells using primers 1) primer A & primer 2, 2) primer B & primer 3, 3) primer A & primer B. 4) Colony PCR for recombinant CusB cells using CusB 5′ & CusB 3′. 5) Colony PCR for endogenic CusB cells (BL21) using CusB 5′ & CusB 3′. 6) Colony PCR for ∆CusB cells using CusB 5′ & CusB 3′. B. Sketch describing fragment sizes for the latter colony PCRs.

Table F: Primers used for mutant formation

mutant	primer
MET11ILE	ATCGGCAGCATCATCGCGGGCGG
MET49ILE	AATCTTATTCTGGTACGACCCAATCTATCCCAATACGCGGTTCG
MET64ILE	TAAACCAGGTAAATCGCCGTTTATCGATATGGATCTGGTGCCGAAATATGC
MET66ILE	TAAACCAGGTAAATCGCCGTTTATGGATATCGATCTGGTGCCGAAATATGC
MET190ILE	ACTGGCGGGAATCCCGGAGGCGGATATTCG
MET227ILE	CGCGGGAATCAATATCGCC
MET241ILE	AAATTCAGGGTATCGACCCGGTGTGG
MET311ILE	AAACCGGGCATCAACGCCTGGTTGC
MET324ILE	AGCGAACCGATCCTGCTCATTCC
MET398ILE	GGTAGCACTTTCAGAGCGGATCCGCTCC

Primers were cloned into the pYTB12 vector using the free ligation PCR technique. Those constructs, which encode for the mutated fusion protein composed of CusB, intein, and a chitin-binding domain, were transformed into the *E. coli* strain BL21 (DE3). Mutants were verified by sequencing (Hy-labs, Rehovot Israel) against the primers: **A.** T7-Terminator (Hy-labs primer) **B.** CusB 5' GTTGTACAGAATGCTGGTCATATGAAAAAAATCGCGCTTATTATCG **C.** CusB 3' GTCACCCGGGCTCGAGGAATT TCAATGCGCATGGGTAGC

Growth rates experiments

To determine the growth-rate dependence on endogenic CusB (native CusB), recombinant CusB (overexpressed CusB), ∆CusB, and cells with empty PYTB12 plasmid, cells were grown in 10 ml LB+ ampicillin (AMP; 10 µl) for 16 hr at 37 °C. Cells were transferred and evaluated in 96-well microplates in dilution of 0.015 (V_{cells} : V_{medium}) for a final concentration of 200 µl LB+AMP medium. Cells were grown for 16 hr at 37 °C without any shaking. Absorbance at 600 nm was measured every 30 min after shaking for 5 s in a 96-well plate reader (SPL Life Sciences, Gyeonggi, Korea). The buffer background was auto-subtracted. Each experiment was repeated five times under identical conditions.

Mutant growth rates were evaluated under similar conditions as described above. The effect of the growth media was studied in 200 µl of a rich medium comprising LB medium with AMP, and in 200 µl of a poor medium comprising M9 medium with AMP. Either Cu(II) (copper(II) chloride (Sigma-Aldrich, St. Louis, MO, USA) or Cu(I) (tetrakis (acetonitrile) copper(I) hexafluorophosphate (Sigma-Aldrich) at various concentrations were added to the growth media.

Each experiment was repeated five times under identical conditions. The presented results are for M9 medium unless indicated otherwise.

As was already reported, endogenic CusB presents a growth rate similar to ΔCusB (7-11). Over-expressed CusB (recombinant CusB) presents higher growth rate compared with ΔCusB.

Growth rate experiments in rich medium

To determine the effect of the medium on ΔCusB-mutant cell growth, LB broth was used as a rich medium for comparison of the growth rates of native *E. coli* cells, and of ΔCusB and ΔCusB-mutants. This set of experiments was also conducted to observe the growth-rate dependence on the concentrations of Cu(II).

Cell viability

Cells were dyed using the L7007 LIVE/DEAD Bacterial Viability kit (Molecular Probes, Oregon, USA); cells were grown in M9 medium. Images were acquired on a Leica SP8 confocal microscope, running LASX acquisition software. The magnification used was a 63 X 1.4 NA objective. Cells were counted with ImageJ software, with a script fully described below. Error calculations were based on three repetitions (Standard deviation), with each experiment scanned at nine different tiles — all together 27 repetitions.

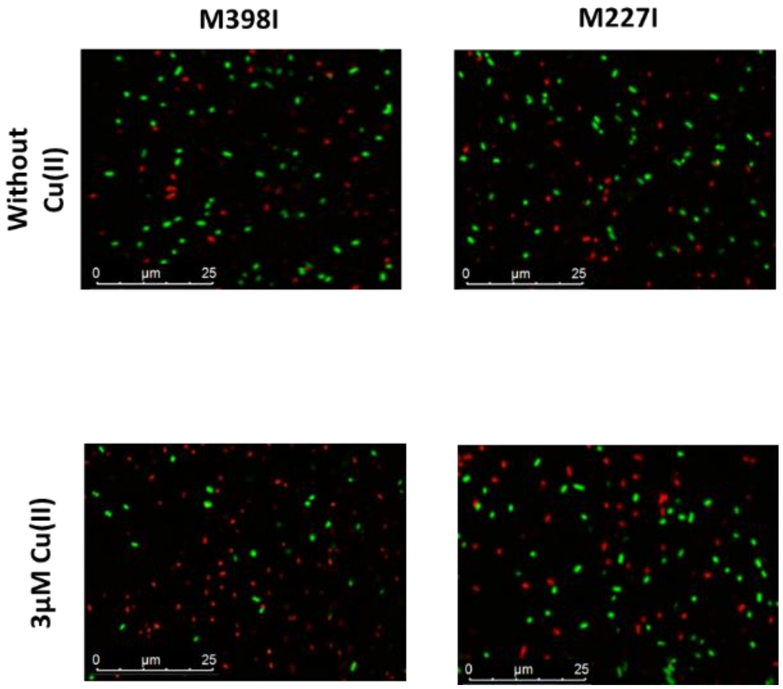

Fig H: CusB mutants: cell viability imaging. Cell viability images for M398I and M227I clones grown with Cu(II) to the late lag phase (3.5–4 hr). Green dots represent live cells, red dots dead cells.

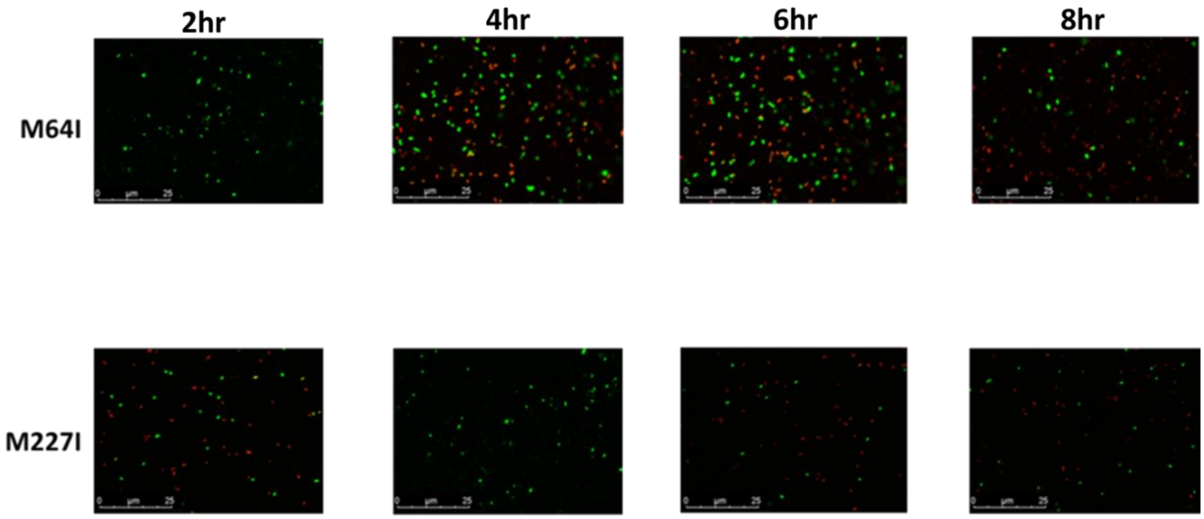

Fig I: CusB mutants: time-dependent cell viability imaging Time dependence of cell viability images of M64I and M22I clones grown with Cu(II). Green dots represent live cells, red dots dead cells.

Cell counting was carried out using ImageJ software, with the following script:

```
// choose the files

//green

green_path = File.openDialog("Choose green File");

green= File.getName(green_path);

dir= File.getParent(green_path);

open(green_path);

//split channels

run("Split Channels");

close();

selectWindow(green + " (red)");

close();

selectWindow(green + " (green)")

setAutoThreshold("Otsu dark");

setOption("BlackBackground", false);

run("Convert to Mask", "method=Otsu background=Dark calculate");

run("Set Measurements...", "limit display decimal=1");

run("Analyze Particles...", "size=0.002-infinity  show=Outlines display clear include summarize add stack");

//print(blue);

saveAs("Tiff", green_path+green+ "_drawing");

selectWindow("Summary of "+ green + " (green)" );

saveAs("Results", dir+"\\green Results "+ green+ ".xls");


// choose the files

//red

red_path = File.openDialog("Choose red File");

red= File.getName(red_path);

dir= File.getParent(red_path);

open(red_path);

//split channels

run("Split Channels");
```

```
close();

close();

selectWindow(red+ " (red)");

run("Median...", "radius=3 stack");

setThreshold(55, 255);

//setAutoThreshold("Otsu dark");

//setOption("BlackBackground", false);

run("Convert to Mask");//, "background=Dark calculate");

run("Set Measurements...", "limit display decimal=1");

run("Analyze Particles...", "size=0.002-infinity  show=Outlines display clear include summarize add stack");

//print(blue);

saveAs("Tiff", red_path+red+ "_drawing");

selectWindow("Summary of "+ red + " (red)" );

saveAs("Results", dir+"\\red Results "+ red+ ".xls");
```

References

1. Meir A, Abdelhai A, Moskovitz Y, & Ruthstein S (2017) EPR Spectroscopy Targets Structural Changes in the E. coli Membrane Fusion CusB upon Cu(I) Binding. *Biophys. J.* 112(12):2494-2502.
2. Don W, Fischer SG, Kirschner MW, & Laemmli UK (1971) Peptide mapping by limited proteolysis in sodium dodecyl sulfate and analysis by gel electrophoresis. *J. Biol. Chem.* 252:1102-1106.
3. Peterson GL (1997) A simplification of the protein assay method of Lowry et al. which is more generally applicable. *Anal. Biochem.* 83:346-356.
4. Jeschke G (2007) *Deeranalysis 2006: Distance measurements on nanoscopic length Fscales by pulse ESR* (Springer) pp 287-288.
5. Jeschke G (2012) DEER Distance measurements on proteins. *Annu. Rev. Phys. Chem.* 63:419-446.
6. Smith PK*, et al.* (1985) Measurement of protein using bicinchoninic acid. *Anal. Biochem.* 150:76-85.
7. Franke S, Grass G, Rensing C, & Nies DH (2003) Molecular analysis of the copper-transporting efflux system CusCFBA of Escherichia coli. *J. bacteriology* 185(13):3804-3812.
8. Outten FW, Huffman DL, Hale JA, & O'Halloran TV (2001) The independent cue and cus systems confer copper tolerance during aerobic and anaerobic growth in Escherichia coli. *J. Biol. Chem.* 276(33):30670-30677.
9. Bagai I, Liu W, Rensing C, Blackburn NJ, & McEvoy MM (2007) Substrate-linked conformational change in the periplasmic component of a Cu(I)/Ag(I) efflux system. *J. Biol. Chem.* 282(49):35695-35702.

10. Oh D, Yu Y, Lee H, Wanner BL, & Ritchie K (2014) Dynamics of the serine chemoreceptor in the Escherichia coli inner membrane: a high-speed single-molecule tracking study. *Biophys. J.*106(1):145-153.
11. Santiago AG*, et al.* (2017) Adaptor protein mediates dynamic pump assembly for bacterial metal efflux. *PNAS* 114(26):6694-6699.
