# Supplementary material for: Exploring the role of the various methionine residues in the Escherichia coli CusB adapter protein
Source: PLoS One. 2019 Aug 29;14(8):e0219337. doi: 10.1371/journal.pone.0219337 (PMC6715271; doi:10.1371/journal.pone.0219337)
Supplement: Attachment — Submitted filename: jb PLOS ONE REF.docx [file pone.0219337.s008.docx]

Developing novel drugs to eliminate new pathogenic bacteria is one of the highest priority in antibiotics research. One of the best approaches is to inhibit copper transporters in prokaryotic systems which is required for many crucial biological pathways. Pathogenic systems contain a highly sophisticated copper-regulation network. Recently, it was revealed that the CusB protein part of the CusCBA periplasmic Cu(I) efflux system in Gram-negative bacteria, play an important key role in the functioning of the whole CusCBA system

In this manuscript, the authors targeted two methionine residues (M227 and M241) that are essential for the proper function of CusB of *Escherrichia coli*. The authors used a combination of in-vitro spectroscopic measurements and cell experiments to study the significance of each single methionine residue in CusB and its effect on cell survival. Indeed, they used pulsed EPR spectroscopy to study the conformational changes of CusB associated with Cu(I) binding.

The combination of the *in-vitro* structural measurements comprised of double electron-electron resonance (DEER) spectroscopy, coupled with site directed spin labeling (SDSL). Also, the DEER technique pulsed electron double resonance (PELDOR) was used to measure the dipolar interactions between two or more electron spins. The authors compared the DEER data with the structure of CusB and used multiscale modeling of macromolecular systems.

Moreover, the authors evaluated the binding affinity of CusB proteins for Cu(I) by using the spectrophotometric competition assay with bicinchoninic acid (BCA) as a ligand. Furthermore, the authors conducted live/dead fluorescence cell imaging experiments to determine whether point mutations might lead to cell death or only to a reduction in *E. coli* cell growth.

In this excellent rationale, they showed that beside essential methionine residues reported before, there were two additional methionine residues at CusB domains 2 and 3 (M227 and M241) that are important for proper function of the CusCBA transporter. Mutating these methionine residues inhibits conformational changes in CusB in the presence of Cu(I) and results in a non-functional CusB protein, decreased copper resistance, and increased cell toxicity in pathogenic cells.

This manuscript is very well written and it was a pleasure reading it. The biochemical analytical work and associated single-point mutation genetics is outstanding.

Tow minor comments are indicated:

1. There are lots of scientific abbreviation like: multicopper oxidase CueO, CopA, CusCBA that should be defined for the non- biochemistry expert.
2. In page	,lines 71 and 72 you have written the following;

The inner and the outer membrane proteins are connected by a linker protein, CusB, in the periplasm, and are at a CusA: CusB: CusC oligomerization ratio of 3:6:3 [16].

QUERY: Do you mean that the inner and the outer membrane proteins are connected by a linker protein, CusB, in the periplasm, which is composed of a CusA: CusB: CusC oligomerization ratio of 3:6:3 [16].

If that’s what you mean, than my correction seems better
